# Evaluation of the Effects of Implants with Different Geometries on the Inferior Alveolar Nerve Under Occlusal Stresses

**DOI:** 10.3390/jfb17060283

**Published:** 2026-06-06

**Authors:** Dilan Karahan, İhsan Çağlar Çinar

**Affiliations:** 1 Oral Implantology Program, Institute of Graduate Studies in Health Sciences, Istanbul University, 34116 Istanbul, Turkey; 2 Department of Oral Implantology, Faculty of Dentistry, Istanbul University, 34116 Istanbul, Turkey; cinarcaglar@istanbul.edu.tr

**Keywords:** dental implant, inferior alveolar nerve, finite element analysis

## Abstract

Dental implant placement in the posterior mandible can be challenging due to the presence of the inferior alveolar nerve (IAN). Biomechanical factors related to implant design and loading may also influence nerve compression. This study aimed to investigate the influence of implant geometry, diameter, length, and implant–canal distance on IAN pressure. Dental implants with two geometries (tapered and cylindrical), two lengths (8 and 12 mm), and two diameters (3.3 and 4.1 mm) were virtually positioned at three implant–canal distances (0.5, 1.0, and 1.5 mm). A total of 24 finite element models were generated. Functional occlusal loading was simulated using a 300 N vertical force and a 100 N oblique force applied at a 45° angle. The resulting IAN pressure values were determined. The implant diameter affected IAN pressure, with 3.3 mm implants yielding higher values than 4.1 mm implants. Longer implants exhibited lower pressure values than shorter implants. Cylindrical implants generated higher pressure than tapered implants. Increasing the implant–canal distance from 0.5 to 1.5 mm reduced nerve pressure. Vertical loading yielded higher pressure values than oblique loading. The implant–canal distance was the primary factor influencing IAN pressure. Implant diameter and geometry had secondary effects, whereas implant length had a limited influence. These findings highlight the importance of implant planning and design selection to reduce load transfer to the IAN.

## 1. Introduction

Dental implant therapy has become a predictable and widely accepted treatment option for the rehabilitation of missing teeth in contemporary dental practice, supported by favorable long-term clinical outcomes and high survival rates [1]. Anatomical structures represent key considerations during implant placement and may impose significant limitations, particularly in the posterior mandible. The inferior alveolar nerve (IAN) is one of the most important anatomical landmarks among these structures. Injury to this nerve may result in substantial neurosensory complications significantly impacting the patient’s quality of life [2,3]. Such outcomes underscore the necessity of accurate preoperative planning, meticulous surgical technique, and comprehensive knowledge of mandibular anatomy [4].

The IAN courses through the mandibular canal and provides sensory innervation to the mandibular teeth, lower lip, and chin region [5,6,7]. Iatrogenic injury to the IAN during implant placement may result in a spectrum of neurosensory disturbances, including hypoesthesia, paresthesia, anesthesia, and dysesthesia [4,8].

Advances in three-dimensional (3D) imaging, particularly cone-beam computed tomography, have substantially improved visualization of the mandibular canal and facilitated more precise spatial planning in implant dentistry [4,9,10]. Nevertheless, the concept of a universally safe implant–canal distance remains debated. A minimum safety distance has traditionally been recommended. However, the biomechanical behavior of neural structures in relation to varying implant–canal proximities under functional loading conditions still remains unclear [11,12].

In addition to anatomical and surgical considerations, occlusal forces transmitted through dental implants may also influence the biomechanical response of the mandibular canal and the enclosed nerve by generating stress and deformation within the peri-implant bone and surrounding mandibular structures. Finite element investigations have demonstrated that implant-related variables, such as implant length, diameter, geometry, and loading direction, significantly influence stress distribution patterns within the mandible [13,14].

Finite element analysis (FEA) has therefore emerged as a valuable methodological approach for evaluating biomechanical behavior under controlled conditions. Previous studies have investigated stress transmission toward the mandibular canal and IAN, highlighting the influence of implant positioning and loading conditions on neural structures [15,16]. Despite this growing body of research, studies specifically quantifying IAN pressure under systematically varied implant design and positional parameters are still scarce.

Therefore, this study was conducted to evaluate IAN pressure using FEA under vertical and oblique loading conditions, with particular emphasis on implant geometry and positional variables. The study specifically aimed to investigate how implant-related variables influence stress transmission toward the inferior alveolar nerve.

## 2. Materials and Methods

### 2.1. Ethical Approval

This study was conducted in collaboration between the Faculty of Dentistry, Istanbul University, and Tinus Technologies. Financial support was provided by the Scientific Research Projects Coordination Unit of Istanbul University under project number TDK-2024-41420. Ethical approval for the study was obtained from the ethics committee of the Faculty of Dentistry, Istanbul University (Approval No.: 2024/61).

### 2.2. Three-Dimensional Model Design

In this study, 3D finite element models were developed, and the corresponding stress analyses were performed. The mandibular bone geometry was generated using tomographic computed imaging data processed to obtain anatomically representative bone structures. The tomographic datasets were reconstructed with a slice thickness of 0.33 mm and imported into the 3D Slicer v5.6 software in Digital Imaging and Communications in Medicine (DICOM) format. The Computed Tomography (CT) data in DICOM format were segmented in 3D Slicer software based on appropriate Hounsfield unit values determined according to the bone density of the posterior mandibular region (threshold range: 426.50–3193.04) and transformed into Standard Tessellation Language format.

**Figure 1:**
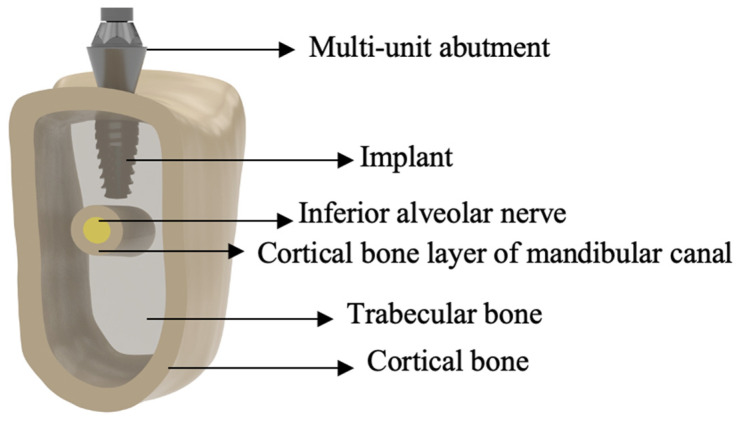
Schematic illustration of the anatomical and structural components of the posterior mandibular model, including the implant, IAN, and surrounding bone tissues.

A cortical bone layer was defined, and the trabecular bone structure was subsequently generated based on the internal surface of the cortical bone (Figure 1). The mandibular region corresponding to the first molar area (tooth #36) was isolated and prepared for FEA. The material properties of the posterior mandibular bone were defined based on the values reported in previous validated FEA studies [17]. Reverse engineering procedures and 3D computer-aided design (CAD) modifications were performed to refine the geometries and ensure proper alignment of all components using Rhinoceros 3D 8 software (Robert McNeel & Associates, Seattle, WA, USA).

The mandibular canal was anatomically identified and delineated within the reconstructed mandibular CT dataset during the segmentation process in 3D Slicer software based on anatomical continuity and radiographic boundaries of the canal structure. Following anatomical delineation, the mandibular canal was standardized and modeled as a cylindrical structure with a diameter of 2 mm and surrounded by a cortical bone layer with a uniform thickness of 1 mm to ensure consistency among the finite element models. Similar to the complex nerve modeling approach described by Sammartino et al. [16], the IAN was modeled as a distinct anatomical structure positioned concentrically within the mandibular canal (Figure 1). This nerve-oriented modeling approach allowed direct evaluation of the pressure transmitted from the implant–bone complex to the nerve. In the present study, “IAN pressure” was used as a comparative biomechanical parameter representing the mechanical load transfer and compression tendency occurring around the inferior alveolar nerve in relation to implant–canal distance and implant-related variables. The reported values were calculated from the mechanical response generated on the modeled inferior alveolar nerve structure within the mandibular canal under the defined loading conditions.

Dental implants with two different geometries (tapered and cylindrical), two lengths (8 and 12 mm), and two diameters (3.3 and 4.1 mm) were modeled and positioned within the mandibular bone. The 3D CAD models of the implants were created using Rhinoceros 3D software based on the manufacturer’s catalog data. Both implant groups presented a thread pitch of 0.7 mm and reverse buttress-like thread morphology. However, the tapered implants exhibited a more aggressive macrogeometry with self-cutting apical features, whereas the cylindrical implants demonstrated a more uniform thread configuration. For each implant configuration, abutments with a diameter of 4.5 mm and a gingival height of 3.5 mm were modeled together with the corresponding fixation screws. Metal substructures and prosthetic crowns were designed to be compatible with the selected abutment systems and positioned to ensure proper implant–abutment compatibility and effective load transfer.

The relationship between the implant apex and the mandibular canal was defined by adjusting the vertical distance between the apical end of the implant and the cortical bone surrounding the canal. The implant–canal distances of 0.5, 1.0, and 1.5 mm were simulated to assess the influence of implant proximity on the IAN. This systematic configuration enabled comparative evaluation of the biomechanical effects of implant design parameters on the IAN.

**Figure 2:**
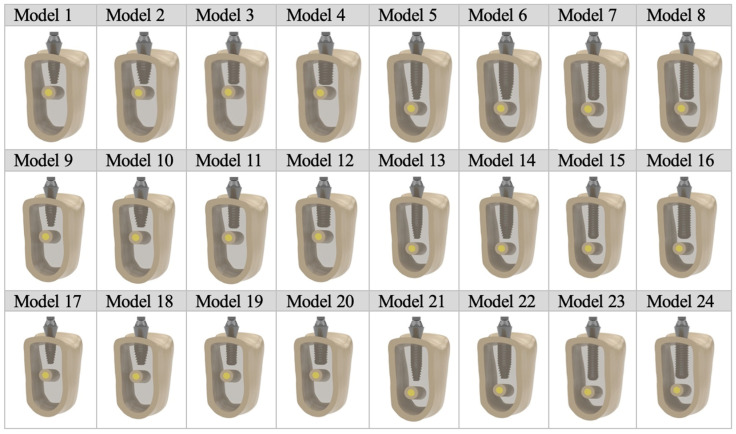
Schematic representation of the different implant configurations used in the finite element analysis.

Based on the combinations of implant length, diameter, geometry, and implant–mandibular canal distance (0.5, 1.0, and 1.5 mm), a total of 24 finite element models were generated for comparative analysis (Figure 2). The finite element models consisted of approximately 288,000–356,000 nodes and 1,170,000–1,448,000 elements depending on the model configuration (Table 1).

Following completion of the modeling process in Blender v4.5 LTS software, finite element mesh structures were generated using Altair HyperMesh v2024 software (Altair, Troy, MI, USA). Surface meshing was performed using high-resolution triangular elements with sizes ranging from 0.1 to 0.25 mm. After surface meshing, the solid geometries were discretized using 3D tetrahedral elements. Proper force transmission between components was enabled by ensuring mesh compatibility between contacting structures using Altair HyperMesh v2024 software. The prepared finite element models were subsequently transferred to the Altair OptiStruct solver (Altair).

A mesh convergence analysis was performed to evaluate mesh sensitivity and improve the numerical consistency of the computational model used in the biomechanical analysis. The primary objective was to determine an appropriate mesh density that would achieve a relative error below 3% while maintaining a balance between computational efficiency and solution accuracy. Accordingly, finite element meshes with varying element sizes ranging from coarse to fine were generated, and all meshes were analyzed under identical loading and boundary conditions to ensure consistency in the comparisons. The variation in the evaluation metric obtained from successive mesh refinements was assessed by comparing the results between consecutive mesh configurations. During model preparation, triangular 2D and tetrahedral 3D meshes were used, and mesh quality controls were performed for all models. Elements with a skewness value greater than 80° or a minimum element length below 0.001 were corrected prior to analysis.

### 2.3. Material Properties

The mechanical properties of the materials, including elastic modulus and Poisson’s ratio, are listed in Table 2.

### 2.4. Loading and Boundary Conditions

**Figure 3:**
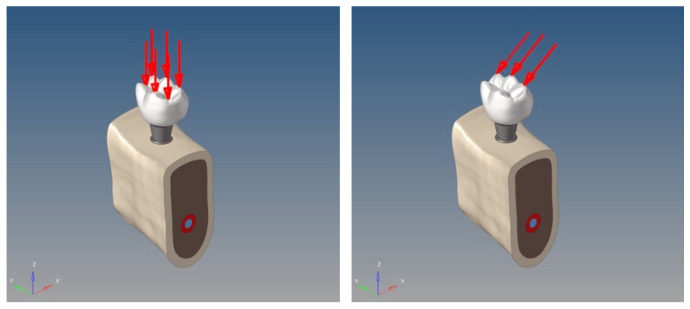
Vertical loading (**left**) and oblique loading (**right**) conditions.

For all models, the loading conditions were defined to simulate occlusal forces acting on the mandibular first molar region. The applied loading conditions were determined based on previous FEA studies [16,20]. A vertical load of 300 N was applied to the central fossa of tooth #36 (Figure 3). Moreover, an oblique load of 100 N was applied to the lingual incline of the buccal cusp of tooth #36 at an angle of 45° (Figure 3). Stress singularities were prevented by distributing the applied loads over multiple surrounding nodes rather than applying them at a single point.

Boundary conditions were established by fully constraining the nodes located in the mesial and distal regions of the cortical and trabecular bones. All translational degrees of freedom along the three spatial axes were restricted to prevent rigid-body motion of the models.

In order to perform the analyses and obtain accurate results in the generated finite element models, the contact relationships between the components constituting the model were defined within the analysis software. Fully bonded contact conditions were assigned to all interfaces, including the implant–bone, implant–abutment, abutment–crown, cortical–trabecular bone, and mandibular canal–nerve interfaces. This modeling strategy assumes complete osseointegration and ideal mechanical continuity among assembled components under functional loading conditions.

A total of 48 linear static analyses were performed for the 24 finite element models under the defined loading and boundary conditions.

## 3. Results

The IAN pressure values were successfully obtained for all finite element models under both vertical and oblique loading conditions. The resulting data are presented in Table 3.

**Figure 4:**
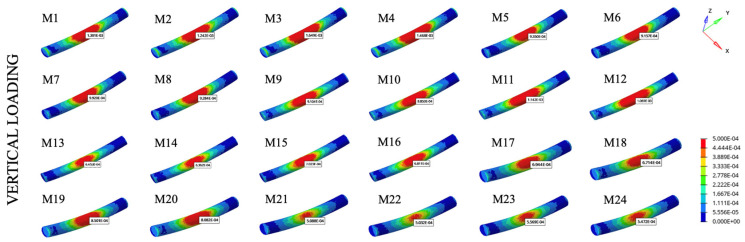
Pressure distribution in the IAN under vertical loading.

**Figure 5:**
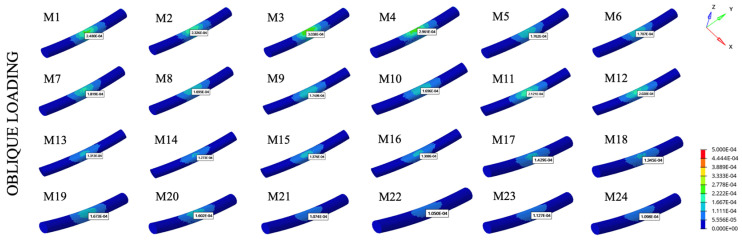
Pressure distribution in the IAN under oblique loading.

The IAN pressure values ranged from 0.0005032 to 0.001649 MPa under vertical loading and from 0.000105 to 0.0003038 MPa under oblique loading (Table 3 and Figure 4 and Figure 5). In all analyzed configurations, vertical loading resulted in higher IAN pressure values than oblique loading.

The highest IAN pressure values, representing the maximum pressures observed under both vertical and oblique loading conditions, were recorded in Model 3, which featured an 8 mm implant length, a 3.3 mm diameter, cylindrical geometry, and a minimal implant–mandibular canal distance of 0.5 mm. On the contrary, the lowest IAN pressure values, corresponding to the minimum pressures observed, particularly under oblique loading conditions, were recorded in Model 22, which featured a 12 mm implant length, a 4.1 mm diameter, tapered geometry, and a maximum implant–canal distance of 1.5 mm (Table 3 and Figure 5).

Comparisons among identical implant configurations placed at different implant–canal distances demonstrated a decrease in IAN pressure values with increasing distance. For example, cylindrical 3.3 mm diameter and 8 mm length implants placed at 0.5 mm (Model 3), 1.0 mm (Model 11), and 1.5 mm (Model 19) distances showed progressive reductions in IAN pressure.

Longer implants generally exhibited lower IAN pressure values than shorter implants, particularly under vertical loading conditions; for instance, when comparing Model 3 with Model 7 and Model 11 with Model 15. Lower pressure values associated with implant length were observed in models with implant–canal distances of 1.0 and 1.5 mm. Under oblique loading conditions, reductions in IAN pressure values associated with implant length were also observed; however, these reductions were smaller than those observed under vertical loading conditions, particularly in narrow-diameter cylindrical implant models, including Model 3 versus Model 7, Model 11 versus Model 15, and Model 19 versus Model 23. Further, longer implants showed lower IAN pressure values in wide-diameter and tapered implant models, such as Model 4 versus Model 8 and Model 12 versus Model 16.

The implant diameter influenced IAN pressure values, with 3.3 mm diameter implants consistently generating higher pressures than 4.1 mm diameter implants across otherwise identical configurations.

When implant diameter, implant length, and implant–canal distance were kept constant, cylindrical implants generally generated higher IAN pressure values than tapered implants under both vertical and oblique loading conditions. This difference was observed in narrow-diameter (3.3 mm) configurations, including Model 1 versus Model 3, Model 9 versus Model 11, and Model 17 versus Model 19, as well as in wide-diameter (4.1 mm) implant models, including Model 2 versus Model 4, Model 10 versus Model 12, and Model 18 versus Model 20.

However, an exception was observed in the comparison between Model 6 and Model 8 under oblique loading conditions: the tapered implant configuration exhibited higher IAN pressure values than the corresponding cylindrical implant under otherwise identical conditions.

## 4. Discussion

The present FEA study provides a controlled biomechanical evaluation of IAN pressure under vertical and oblique loading conditions, with specific emphasis on implant-related design and positional parameters. Implant system characteristics were standardized, and implant length, diameter, geometry, and implant–canal distance were systematically varied to isolate the key determinants of load transmission to the mandibular canal and contextualize these findings within both biomechanical and clinical literature.

Across all model groups, the implant–canal distance emerged as the primary determinant of IAN pressure. Models representing minimal implant–canal distances (Models 1–8) consistently demonstrated higher IAN compared with models incorporating increased separation from the mandibular canal (Models 17–24), regardless of implant length, diameter, or geometry. This inverse relationship between implant proximity and nerve pressure was preserved under both vertical and oblique loading conditions and was consistent with earlier FEA investigations reporting a steep increase in neural compression with a decrease in apical distance [15,16]. Clinical radiological studies further reinforce these biomechanical findings by reporting more favorable neurosensory recovery outcomes when implants are positioned close to, but not in direct contact with, the mandibular canal, or classified as “close” or “separate” rather than intruding into the canal [21]. Taken together, the present model-based findings and previous clinical observations suggest that implant–canal distance may play an important role in neural stress transmission.

Implant length demonstrated a comparatively limited effect on IAN pressure. When implant geometry, diameter, and implant–canal distance were held constant, longer implants generally exhibited lower IAN pressure values than shorter implants, particularly under vertical loading conditions. Comparisons between short and long implants within identical implant–canal distance groups indicated that increased implant length was associated with lower nerve pressure under vertical loading conditions. However, this advantage diminished under oblique loading and became less pronounced with an increase in implant–canal distance. This observation was consistent with previous FEA studies reporting that implant length alone did not govern stress transmission to adjacent anatomical structures when other geometric variables were controlled [16]. This finding may be explained by the increased bone–implant contact area provided by longer implants, which may facilitate load distribution over a broader area and thereby reduce localized stress concentrations around the mandibular canal. From a clinical perspective, the influence of implant length on stress transmission toward the IAN appeared limited.

Implant geometry significantly influenced IAN pressure distribution patterns in a configuration-dependent manner. In a majority of matched configurations, cylindrical implants generated higher IAN pressure values than tapered designs under identical conditions. This trend is biomechanically plausible, as cylindrical implants tend to transmit higher shear components, whereas tapered implants promote more favorable compressive load dissipation along the implant body [22]. In addition, tapered implants may facilitate a more gradual transfer of occlusal forces to the surrounding bone due to their convergent macrogeometry, thereby reducing localized stress accumulation around the mandibular canal. In contrast, the parallel walls of cylindrical implants may contribute to a less favorable stress distribution pattern and greater concentration of stress within the peri-implant region.

However, isolated configurations demonstrated deviations from this general trend, indicating that the biomechanical effect of implant geometry on neural loading was not uniform across all model combinations. Such discrepancies may be attributed to complex interactions among implant geometry, diameter, implant–canal distance, and loading direction, which can alter local stress trajectories and bending moment distributions within the peri-implant region. Therefore, the influence of implant geometry on IAN pressure should not be interpreted in isolation but rather in conjunction with other geometric and positional parameters. Although clinical trials have reported comparable survival and success rates between tapered and cylindrical implants, tapered designs often exhibit more favorable primary stability characteristics during early healing [23], potentially supporting their biomechanical advantage under complex loading scenarios.

The implant diameter exhibited a consistent modulatory effect on IAN pressure across all implant–canal distance groups. Models incorporating narrower-diameter implants (Ø 3.3 mm) demonstrated higher IAN pressure compared with corresponding wider-diameter configurations (Ø 4.1 mm) under identical conditions, irrespective of implant length or geometry. This observation was consistent with biomechanical findings indicating that increased implant diameter enhances structural rigidity and reduces stress concentration in surrounding bone [24]. From a neuromechanical perspective, the reduced peri-implant deformation associated with wider implants may attenuate pressure transfer toward the mandibular canal, particularly under oblique loading conditions that amplify stress gradients. In addition, the lower pressure values observed in wider-diameter implants may also be associated with a more favorable stress distribution pattern, as occlusal forces are dispersed over a broader implant–bone contact area, thereby reducing localized stress concentrations under both vertical and oblique loading conditions.

Comparative percentage-based evaluations performed using matched model pairs with otherwise identical implant configurations demonstrated that implant–canal distance exerted the strongest influence on calculated IAN pressure values, followed by implant length and implant geometry, whereas implant diameter demonstrated the smallest influence across the evaluated model groups. Furthermore, progressive reductions in IAN pressure values were consistently observed with increasing implant–canal distance under both vertical and oblique loading conditions. Cylindrical implants tended to generate higher IAN pressure values than tapered implants in most matched comparisons, whereas longer implants tended to reduce IAN pressure values more markedly under vertical loading conditions.

Beyond studies directly addressing IAN proximity, a substantial body of FEA studies has investigated the influence of implant length, diameter, bone density, and loading direction on mandibular biomechanics. Although these studies primarily focused on peri-implant bone or implant component stress rather than neural structures, their findings provide important contextual support for the neuromechanical trends observed in the present study. Previous implant biomechanics studies have generally reported higher stress concentrations under oblique loading conditions compared with vertical loading [25]. However, these studies primarily evaluated peri-implant bone stress rather than stress transfer toward neural structures. In the present study, lower IAN pressure values were observed under oblique loading conditions. This finding may be related to the tendency of implants to undergo greater bending in the cervical region under oblique loading, resulting in stress concentration around the implant neck and reduced stress transmission toward the apical region and the IAN.

The relevance of a standardized “safety distance” between the implant apex and the mandibular canal remains controversial. Previous biomechanical studies proposed a minimum distance of approximately 1.5–2.0 mm to reduce the risk of nerve compression, whereas large retrospective clinical analyses demonstrated that implants placed within 2 mm of the mandibular canal did not necessarily result in neurosensory complications [26,27]. These clinical observations showed similarities with the trends observed in the present findings, in which all calculated IAN pressure values remained below the proposed functional conduction block threshold. Experimental studies have suggested that sustained pressures in the range of 100–200 mm Hg (≈0.013–0.026 MPa) may induce temporary peripheral nerve conduction block without structural damage [16,28]. In this context, this pressure range represents a functional threshold associated with reversible alterations in nerve conduction rather than a definitive indicator of permanent neural injury. The proposed values are derived from experimental conditions involving prolonged, uniformly applied compression to peripheral nerves and therefore should be interpreted as the reference levels for functional impairment rather than absolute injury limits. In this context, the consistently lower IAN pressure values observed across all models in the present study suggest that the calculated pressure values were lower than the experimentally reported reference ranges associated with transient conduction alterations under sustained loading conditions.

Although all calculated IAN pressure values remained below the experimentally reported reference ranges associated with transient neural conduction alterations, relative differences among implant configurations were consistently observed within this sub-threshold biomechanical range. Therefore, the proposed threshold values may serve as a useful neurophysiological reference for contextualizing the calculated pressure values, while the comparative differences among models provide insight into the relative influence of implant-related variables on stress transmission toward the IAN.

A comparison with previous studies revealed that the nerve pressure values reported by Sammartino et al. differed from those obtained in the present study. These discrepancies can be primarily attributed to the differences in modeling strategies and loading conditions. In the study by Sammartino et al., the prosthetic superstructure was not incorporated into the model, and loads were directly applied to the implant–abutment complex. This simplification likely resulted in a more localized and concentrated transfer of forces, potentially leading to increased stress transmission toward the IAN [16].

A similar trend was observed in the study by Gasparro et al., in which the bone structure was simplified into a cylindrical block. This geometric idealization might have altered the global mechanical response of the model and influenced the load transfer mechanism. Moreover, the lack of a detailed anatomical representation of the cortical layer surrounding the IAN might have allowed the transmission of loads more directly to the nerve, potentially contributing to higher pressure values [28].

In contrast, the present study employed an anatomically based modeling approach in which the IAN was explicitly enclosed within a cortical canal structure. This configuration enabled a more physiologically relevant load transfer pathway, whereby forces were first distributed within the surrounding bone and the cortical layer of the mandibular canal before reaching the nerve. Consequently, this intermediate structure acted as a biomechanical buffer, attenuating the transmitted load and resulting in lower pressure values compared with previously reported values while also providing a more consistent reduction in nerve pressure with increasing implant–nerve distance [16,28].

Clinical investigations further indicate that most neurosensory disturbances following mandibular implant placement are transient and resolve over time, even in cases of close canal proximity [21,29]. This interpretation is further supported by clinical radiological studies reporting the absence of neurosensory disturbances in implants placed at distances well below the traditionally recommended 2 mm safety zone, provided that direct invasion or compression of the IAN does not occur. Notably, in these studies, the implants associated with uneventful neurosensory outcomes were frequently positioned at mean implant–canal distances of approximately 0.75 mm, indicating that smaller implant–canal distances have also been reported without neurosensory complications [12]. Taken together, these findings suggest that neural integrity is influenced by a multifactorial interplay of mechanical, surgical, and biological factors.

Within the limitations of this finite element model, the findings reinforce implant–canal distance as the principal determinant of IAN pressure, whereas implant length, diameter, and geometry exert secondary, yet biomechanically relevant, influences. The present study was based on a purely computational model and did not include experimental, clinical, or patient-specific validation, representing an important limitation of the study. In addition, patient-specific anatomical variability, including differences in cortical bone thickness, bone density, mandibular canal morphology, and trabecular bone structure, was not incorporated into the model. Therefore, the present findings should primarily be interpreted as biomechanical observations regarding stress transmission toward the IAN rather than direct predictors of clinical neurosensory outcomes. In addition, the IAN was modeled as a simplified linear-elastic material, which does not fully replicate the complex behavior of neural tissues under physiological conditions. However, this assumption has been widely adopted in previous finite element studies investigating implant-related stress transmission around the mandibular canal, thereby allowing standardized biomechanical comparisons among different model configurations. Therefore, the calculated pressure values should be interpreted primarily as relative comparative indicators among model configurations rather than absolute predictors of neural injury or clinical neurosensory response [17]. Future studies incorporating patient-specific bone properties, dynamic loading protocols, and ultra-short implant designs may further refine the predictive value of biomechanical modeling for neurosensory risk assessment in the posterior mandible.

## 5. Conclusions

In this study, implant–canal distance was identified as the dominant factor influencing IAN pressure under both vertical and oblique loading conditions. Increasing the distance between the implant apex and the mandibular canal consistently reduced nerve pressure values. Implant diameter and geometry showed secondary effects, with narrower diameters and cylindrical designs generally associated with higher nerve pressure, whereas implant length demonstrated a limited and configuration-dependent influence. All calculated pressure values remained below the neurophysiological reference threshold reported in the literature. However, this finding should be interpreted cautiously, given the assumptions inherent to finite element modeling. These findings may contribute to a better biomechanical understanding of implant-related stress transmission toward the IAN.

## Figures and Tables

**Table 1:** Description of the models.

Model	Geometry	Length (mm)	Diameter (mm)	Implant–Canal Distance (mm)	Node Count	Element Count
1	Tapered	8	3.3	0.5	288,663	1,172,397
2	Tapered	8	4.1	0.5	307,939	1,248,566
3	Cylindrical	8	3.3	0.5	293,414	1,191,470
4	Cylindrical	8	4.1	0.5	319,857	1,297,304
5	Tapered	12	3.3	0.5	293,308	1,195,296
6	Tapered	12	4.1	0.5	315,262	1,283,680
7	Cylindrical	12	3.3	0.5	299,731	1,218,732
8	Cylindrical	12	4.1	0.5	331,995	1,348,073
9	Tapered	8	3.3	1	299,792	1,221,587
10	Tapered	8	4.1	1	318,816	1,297,710
11	Cylindrical	8	3.3	1	304,777	1,242,119
12	Cylindrical	8	4.1	1	330,163	1,344,249
13	Tapered	12	3.3	1	313,294	1,277,779
14	Tapered	12	4.1	1	336,108	1,369,283
15	Cylindrical	12	3.3	1	318,429	1,295,188
16	Cylindrical	12	4.1	1	351,749	1,429,729
17	Tapered	8	3.3	1.5	287,495	1,170,157
18	Tapered	8	4.1	1.5	307,745	1,251,593
19	Cylindrical	8	3.3	1.5	292,431	1,191,054
20	Cylindrical	8	4.1	1.5	318,076	1,293,688
21	Tapered	12	3.3	1.5	316,741	1,291,627
22	Tapered	12	4.1	1.5	340,308	1,387,080
23	Cylindrical	12	3.3	1.5	323,040	1,314,671
24	Cylindrical	12	4.1	1.5	355,995	1,448,094

**Table 2:** Material properties of the finite element model [17,18,19].

Component	Modulus of Elasticity (MPa)	Poisson’s Ratio
Cortical bone	13,700	0.3
Cancellous bone	1370	0.3
Titanium	115,000	0.35
Trigeminal nerve	1.3	0.4
PoM	73,200	0.3
Metal	240,000	0.29

**Table 3:** IAN pressure values calculated under vertical and oblique loading conditions.

Model	IAN Pressure Vertical Loading (MPa)	IAN Pressure Oblique Loading (MPa)
1	0.001301	0.000248
2	0.001242	0.0002326
3	0.001649	0.0003038
4	0.001468	0.0002961
5	0.000935	0.0001762
6	0.0009157	0.0001707
7	0.000992	0.0001819
8	0.0009284	0.0001695
9	0.0009104	0.0001749
10	0.0008853	0.0001696
11	0.001142	0.0002121
12	0.001069	0.0002028
13	0.0006453	0.0001313
14	0.0006362	0.0001273
15	0.0007023	0.0001376
16	0.0006811	0.0001308
17	0.0006944	0.0001429
18	0.0006714	0.0001345
19	0.0008501	0.0001673
20	0.0008082	0.0001602
21	0.0005088	0.0001074
22	0.0005032	0.000105
23	0.0005569	0.0001127
24	0.0005472	0.0001098

## Data Availability

The original contributions presented in this study are included in the article; further inquiries can be directed to the corresponding author.
